# Pulpit Power

**Published:** 1863-03

**Authors:** 


### PULPIT POWER

Is in "The Word," which "is sharper than a two-edged sword;" which "goeth forth," "conquering and to conquer," and does "not return—void." In all ages, the greatest preachers, those who have accomplished most in "winning souls," were those who had the Scriptures at their fingers' end; whose clearest expositions of the Bible were those which the Bible gave of itself. In Abraham's day, in Jacob's, and later, during the Theocracy in the Wilderness, and on to the times of Peter and Paul, and the more modern orators of the Church, the most powerful addresses were those which were either almost wholly derived from Scripture histories, or from a narration of facts occurring within the memory of the multitude addressed.

The Bible is declared to be "the power—unto salvation;" it is the main instrument in the world's redemption from vice, and its elevation to purity, peace, and immortal life. Bible-reading men do not deny these things in theory, but many there are, too many, alas! who abnegate them in their daily practice; and the custom of the times is tending to a passive rejection of the Sacred Scriptures; not, indeed, by denying their authority, but by letting them go out of sight. Whatever, then, promotes the oblivion of the Bible, is adverse to the best interests of Christianity; and they who are its fastest friends, can never be so well employed as in counteracting these influences, which are kept at work by the arch enemy of man, and by the passions and frailties of our nature.

The tendencies of the times are more and more to fiction, less and less to fact. Fiction, indeed, may have its uses in reasonable bounds, and in judicious hands; but, as an almost universal vehicle of sentiment, it not only weakens the mind, it paralyzes the intellect and debases the heart. The majority of new books are books of fiction. Three-fourths of all the reading matter of the multitudinous periodicals of the hour is admitted fiction, and a very large share of it is the merest namby-pamby, or deals in the wildest exaggerations of all that is degrading in action, vicious in morals, and horrible in fact. Dictionaries are thumbed and worn out in the search of words of the most awful significancy, and these are printed in mammoth letters of black, red, and blue, and are pasted on the sidewalk, against the gutter-stones, on lamp-posts and fences and blind walls, in order to force the attention of the hasty throngers of the streets, to papers, and books, and magazines which are worse than worthless. Within a block of the Bible House, a wall was covered during the Anniversaries with posters, having, among other headings, Adultery, Murder, Blood, Fiends, Ghouls!

These things are not confined to the most degraded newspapers. Wood-cuts by the dozen, in successive months, have been the main feature of weeklies, claimed to be owned by respectable people, and written for by professional men, and purchased by scholars and reputable fathers and mothers, to be read by daughters just emerging into womanhood; pictures representing murders the most atrocious; the brutalizing of almost denuded women; the fac-similes of adulterous confessions, with most disgusting particularities. Not only has the secular press been drawn into this vitiated current of public demand, but portions of the semi-religious and the professedly religious have so far fallen in with the tide, as to devote one or more columns of their weekly issues to the recital of histories, which are no histories at all, of things which never passed as narrated, except in the brains of their imaginative writers.

Nor is this all; this fictitious literature is so prevalent, that it has crept into religious libraries; the fore time "tract," which, as long as it was a verity, was a power in Christendom, is now so loaded down with fictitious, or largely exaggerated narrations, that it is a Samson shorn of the locks of strength. And, more, when Sunday-school books were literal records of actual occurrences, such as the Dairyman's Daughter, and Henry Martin, and Claudius Buchanan, and Harlan Page, their influences for good were precious beyond compare. But who shall say that one in a dozen later "stories" are literally facts? that there are not "fillings in" "to match," which destroy their truthfulness? And what are our "Anniversaries," when compared with those of glorious memories of twenty-five years ago! They are the shell without the kernel; they are the sound without the substance; their platforms are arenas for exaggeration, for effect, for "management," akin to legerdemain, not wholly so, but so in part, too large; in part so large, as to suggest the inquiry in watchful and conservative minds, whether they are not, on the whole, injurious?

In the light of all these things, it is suggested, if it would not be better to come back to the Bible and to plain facts, and make these, to a greater extent, "the weapons of our warfare." And such is the object of some writers, to use historical and Bible facts in the illustration of precepts and principles, which are to guide the conduct and mold the characters of the young of the families into which it may enter. Fact is stranger and stronger than fiction. Why not, then, use it? Bible facts are the strongest of all, because they are whole facts—are true histories, and hence, as vehicles of information and illustration, are immeasurably safer than any others; and, if so, why not seek to store our children's minds with them, instead of the monstrous absurdities of heathen mythology, as contained in "all Greek, all Roman story!" The heroes of the Old Testament, of antediluvian ages, of patriarchal times, of the Theocracy, and onward—there are no stronger characters in all the world's history; they have a beauty and a power belonging to none others—the beauty and the power of truth! Why, then, throw this treasure away, and exchange it for the trash of Homer, and Ovid, and Horace, and Virgil, and then call it a "classical education!" Away with such balderdash, and let a better and a safer era be inaugurated. Let the Bible be the text-book of our children; let its facts, and its incidents, and its veritable histories, be made a part and parcel of their education; and, in due time, we will be a "nation whose God is the Lord," in that it has been "rooted and grounded" in the truth.—

